# Student Perspectives on Campus-Based Mental Health Initiatives: Insights From an Urban Indian Context

**DOI:** 10.7759/cureus.93572

**Published:** 2025-09-30

**Authors:** Seema Mehrotra, Rohit Jaiswal, Sangeeta Menon, Tanvi Agarwal, Manjula Munivenkatappa, Neha Dahiya

**Affiliations:** 1 Clinical Psychology, National Institute of Mental Health and Neurosciences, Bengaluru, IND; 2 Preventive Medicine, Indian Council of Medical Research, New Delhi, IND

**Keywords:** community mental health, community psychiatry, health promotion, mental health, student support services

## Abstract

Introduction

Stress and mental health challenges are common among students, and educational campuses can play an important role in promoting mental well-being. Research on preventive and promotive interventions in educational settings highlights the importance of understanding students’ perspectives and engaging them as key stakeholders. This study explored the views of urban Indian students on strategies to support mental health, including their involvement in program implementation, the role of peer support, and the use of self-help resources.

Methods

We conducted a qualitative, exploratory, descriptive study using focus group discussions (FGDs) to examine student perspectives on themes related to the study objectives. Sixty-nine participants were recruited through purposive sampling from five diverse educational institutions representing both public and private institutes catering to students from grade 9 through undergraduate levels (n = 69; five institutions) in the Bengaluru Urban district of Karnataka state in India. FGDs were audio-recorded and supplemented with detailed field notes. Data were thematically analyzed using a hybrid inductive-deductive approach.

Results

Six themes and 17 subthemes emerged from the analysis of student FGDs. Students reported unmet needs for campus mental health promotion and described stressors including depression, examination-related anxiety, family and societal expectations, bullying, isolation, and difficulty managing negative emotions alongside limited awareness of available help. They called for sensitizing parents and teachers and for training teachers to build connections, recognize distress, and respond supportively. Participants prioritized a supportive campus climate with visible mental health information (e.g., classroom discussions and assemblies), safe spaces, inclusive activities, and dedicated time that reduces academic pressure and enables self-care and skill-building. Students endorsed peer engagement (e.g., training peer champions and strengthening peer support systems) to raise awareness, encourage help-seeking, and support navigation of academic and social challenges. They valued self-help resources but cited barriers to sustained use (e.g., motivation, stigma, and time); they favored interactive, technology-enabled materials and cautious use of social media given risks of distraction and misinformation. Finally, they advocated for formal, easily accessible counseling with clear pathways, strong confidentiality, and individualized care, and expressed mixed views on helplines, balancing the benefits of anonymity against doubts about effectiveness.

Conclusions

This study found that students view campus-based mental health promotion as a priority and identified several essential components for success: sensitization of parents, teacher preparation to recognize and respond to distress, structured peer support, integration of mental health into the academic schedule, supportive environments that reduce stigma, engaging self-help resources enhanced by technology, and confidential, personalized counseling services. Implementing programs that reflect these priorities can foster earlier recognition of distress, increase help-seeking, and improve well-being, offering a practical and culturally relevant approach to addressing youth mental health in educational settings.

## Introduction

Extensive literature addresses stress, distress, and mental health vulnerabilities during adolescence and young adulthood [1-4]. Educational institutions play a critical role in promoting students’ mental health and well-being. India, which has one of the largest education systems in the world, spans both school and higher education and offers numerous opportunities and challenges for implementing effective, scalable, and sustainable mental health programs [5-7]. Recognizing young people’s right to participate in decisions affecting their lives and amplifying their voices in mental health promotion is widely acknowledged as a critical step [8,9]. However, the perspectives of primary stakeholders (i.e., the students themselves) are often underemphasized in the development and implementation of campus-based mental health initiatives [10,11].

An expanding body of research examines preventive and promotive interventions in educational settings [12,13]. Most studies originate from developed countries, underscoring the need to explore implementation strategies that are culturally grounded and adapted to students’ specific contexts [14,15]. A key approach is to incorporate students’ own experiences, perceived needs, and recommendations into program design and implementation [8,11]. This strategy can enhance uptake, acceptability, and satisfaction with mental health programs. Although some studies in India have examined student perspectives to inform the implementation of educational mental health interventions, further research is needed [16-18].

We aimed to explore the perspectives of students in grades 9-12 and undergraduate programs on (i) strategies to support student mental health on campuses; (ii) student involvement in program implementation and peer support; and (iii) usefulness of self-help resources in promoting mental well-being. The study was conducted in Bengaluru Urban District, Karnataka, India, as part of the formative phase of a larger, multistate, national health research priority implementation project on suicide risk reduction and improvement of mental well-being among school and college students, spearheaded by the Indian Council of Medical Research.

## Materials and methods

We employed a qualitative, exploratory, descriptive design using focus group discussions (FGDs) to explore youth perspectives related to the study objectives. Participants were recruited through purposive sampling from five diverse educational institutions representing public and private sectors and serving students from grade 9 through undergraduate levels in the Bengaluru Urban district of Karnataka, India. These institutions included one government school, one private school, a government-aided pre-university institute, one government college, and one private college. All students in grades 9th/10th/11th/12th or studying an undergraduate course were eligible to participate. The study was conducted between June 2024 and July 2024. The selection of participants was subject to institutional permission and feasibility. Purposive sampling was operationalized by inviting students across sections who met the grade-level eligibility, with efforts to ensure representation of all genders. Recruitment was conducted on a first-come, first-served basis, through classroom announcements about the study with the aim of recruiting 10-20 participants per institute. No formal exclusion criteria were applied; however, participation was subject to institutional feasibility, which included factors such as permission to conduct the FGDs, the undergraduate batch selected by the institute (at the college level), availability of students, and logistic support. The period close to holidays or exams was avoided, and the informed consent was sought two to three days in advance to minimize availability bias.

Written informed consent was obtained from participants aged ≥18 years. For those aged <18 years, written assent was obtained along with parental consent. Participation was voluntary, and thus there were no refusals to participate. Five FGDs were conducted, one in each institution, with 13-17 mixed-gender participants per group. The Institutional Ethics Committee approved the study protocol (approval NIMHANS/44th IEC (BEH.SC.DIV.)/2023).

Data collection

An FGD guide was developed by the research staff to address the three interlinked study objectives. Open-ended questions elicited detailed, spontaneous responses, followed by supplementary probes as needed (Table 1). The supplementary probes are available in Appendix A.

**Table 1:** FGD main probes FGD, focus group discussion

Probe	Topic
1	What are the common challenges students face that affect their well-being?
2	What are your views on promoting mental health and well-being in students, as is done for physical health and well-being?
3	Mention a few things that can be done on campuses to promote students’ mental well-being.
4	What role can students themselves play in creating an environment wherein talking about emotional difficulties is not looked down upon?
5	Would it be of any help to make information and self-help tips on various topics (e.g., nature of common mental health problems, coping with stress, and when to go for professional help) available for students?

The first author, with more than 20 years of professional experience, reviewed the probes for clarity and flow. These FGD probes were further reviewed during a meeting of experts from the funding agency before finalization. The guide was prepared in English and Kannada, the official language of Karnataka, with the assistance of a bilingual expert; another bilingual expert cross-validated the Kannada version.

Each FGD was conducted by a trained facilitator, supported by a co-facilitator who took field notes. Discussions took place in relatively quiet spaces within the institutions, such as empty classrooms, lecture halls, or libraries. No teacher/staff was present during the group discussions. Sessions were conducted in the participants’ preferred language (English or Kannada) and lasted 45-60 minutes. Three group discussions were conducted in Kannada and two in English. At the start of each session, facilitators introduced ground rules emphasizing voluntary participation, mutual respect, and non-disclosure of personally identifying information. A preamble explained the study’s purpose and encouraged sharing diverse perspectives rather than reaching consensus. All FGDs were audio-recorded with consent, supplemented by detailed field notes.

Data analysis

Audio recordings were transcribed verbatim in the language of the discussion by the facilitators. Kannada transcripts were prepared by bilingual research staff, then translated into English for coding. A second staff member cross-checked translations. Field notes supplemented transcripts to ensure completeness. Any differences were resolved through discussions within the team.

Thematic analysis followed Braun and Clarke’s six-step framework: familiarization with the data, generation of initial codes, searching for themes, reviewing themes, defining and naming themes, and producing the final report [19]. We used a hybrid inductive-deductive approach, generating a priori codes from the FGD guide while allowing new themes to emerge from the data. Coding was conducted manually, with each FGD independently coded by a research team member.

Dependability was ensured through standardized data collection procedures and detailed documentation of coding processes. Confirmability was supported by regular peer discussions and senior review of the audit trail and analytic process. Themes were refined through constant comparison across groups. Thick descriptions were developed using excerpts and contextual understanding. Discrepancies in coding or interpretation were resolved through collaborative discussion to maintain analytical consistency.

## Results

The basic sociodemographic characteristics of the FGD sample are presented in Table 2. Six themes and 17 subthemes, with corresponding excerpts, are summarized in Table 3. It may be noted that all the subthemes mentioned in Table 3 and described subsequently emerged across two or more FGDs.

**Table 2:** Sociodemographic details of study participants (N = 69) Mean age: 16.79 years; SD: 2.48; median: 17 years

Sociodemographic variable		N (%)
Age (N = 69)	13-17	39 (56.52%)
	18-27	30 (43.48%)
Sex	Male	38 (55.07%)
	Female	31 (44.93%)
Level of study	School (9th and 10th grades)	27 (39.13%)
	Pre-university (11th and 12th grades)	14 (20.29%)
	Undergraduate	28 (40.58%)
Nature of the institute	Government	27 (39.13%)
	Private	42 (60.87%)

**Table 3:** Youth perspective themes, subthemes, and excerpts

Theme	Subthemes	Representative statements
Perceived need for mental health promotion	Experience of stress	“Students experience a lot of pressure during examinations; parents’ expectations add to the pressure.”
		“…then there’s bullying, too. It’s not very nice to corner a single student or to constantly ignore them.”
		“Even when someone has scolded us, we overthink about those things.”
	Lack of mental health initiatives on campuses	“There is focus on physical health through various sports and activities, but no one talks about mental health.”
Need for engaging with parents and teachers	Need for sensitizing parents and teachers on student mental health	“Parents compare us to cousins who may be in better colleges or scoring more marks. Even teachers do that.”
		“When we try to share mental health concerns with our parents, they tell us, ‘We never had all this (in) our time’; instead of understanding, they give us these explanations.”
	Equipping teachers to build personal connections and support students in distress	“If I want to share something with my teacher, I want the teacher to understand it. I want more personal connect(ion) with the teacher.”
		“If I go tell teachers, they usually say, ‘It’s just two years; you’re in first PUC, then you’ll be going somewhere else. So don’t worry about all this.’”
		“If we tell teachers something, they’ll go tell it in front of the entire class.”
		“Teachers should learn to identify students who are struggling and help them.”
Creating a supportive environment for mental health	Enhancing awareness	“There is a need for recognizing that mental health is as important as physical health.”
		“Recognizing that mental health concerns can affect anyone, even people who are busy.”
	Strengthening social connections and inclusivity within campuses	“Educating students to encourage their friends to see everyone as equal and try to include everyone in campus activities will help.”
	Facilitating open conversations on mental health	“Offline community of students can be built where we can discuss mental health on a weekly basis.”
	Allocating dedicated time for mental health	“Dedicate some time exclusively to create awareness about mental health - not like an extra thing.”
	Opportunities to learn and grow	“Students should be encouraged to learn and progress individually depending on their own interests and talents.”
Student engagement and peer support	Usefulness of training student volunteers	“Not only will we get the opportunity to gain new knowledge, but we will also help other people.”
	Peer mentoring system	“Educating students about the impact of teasing and promoting empathy among students can create a supportive environment on the campus.”
		“Train students who are friendly and approachable to spread awareness about mental health.”
Access to resources for students	Barriers to uptake of self-help resources	“Not everyone finds it useful. Self-help resources might not help us. First day we might have motivation to use but after that it will decline.”
		“…because of our busy schedules, we don’t get time to read through mental health materials.”
		“If people see you reading about mental health, they think something must be wrong with you, so many avoid it.”
		“It needs to be sentimental and emotional. What you will show to us needs to be emotional for us to take into our personal lives.”
	Leveraging technology for mental health	“Self-help resources in the form of videos may be impactful.”
		“Use of podcasts, digital platforms like websites, and involving influencers can really help spread awareness about mental health.”
		“We enjoy digital or visual things. We are more engaged in such activities.”
Strengthening mental health services for students	In-campus services for students in need	“There should be a counselor on every campus.”
		“If we have some issues, like a love breakup, we can’t do anything; we’re just upset. We can’t go directly to a professional at our age.”
		“We need an unknown person who will listen and help.”
	Trust as a facilitator for the uptake of counseling services	“It’s easier to open up when one feels that whatever they share is safe and will not be discussed with anyone.”
	Need for individualized support	“Some students are going through a difficult time; talking in front of everyone may not be useful. Personal counselling may help.”
	Role of helplines	“It may work for some people; it may not work for as many people find it easier to talk in person.”
		“Some people are comfortable sharing with people whom they don’t know; it’s anonymous, so that leaves no scope for judgment.”

Theme 1: Perceived need for mental health promotion

Across the FGDs, participants emphasized the need for mental health promotion initiatives on campuses. They described experiencing stressors such as depression, anxiety related to examination performance, pressure and high expectations from families and society, rumination over failures or unmet expectations, interpersonal difficulties including bullying or teasing, social isolation, and challenges in managing negative emotions. Participants reported that mental health issues are often overlooked or inadequately addressed in educational settings and that few initiatives or resources exist to address these needs. As a result, students may feel unsupported and unaware of available help.

Theme 2: Need for engaging with parents and teachers

Participants identified a critical need to sensitize parents and teachers to student mental health issues. They noted that limited awareness and understanding among these key stakeholders often result in inadequate support for students experiencing mental health challenges. Participants observed that parents and teachers may fail to recognize signs of mental distress or appreciate the importance of timely intervention. They recommended educational workshops and informational resources to improve knowledge and strengthen student support.

Participants also stressed the importance of equipping teachers with skills to build personal connections with students and identify those at risk. They reported that teachers trained to recognize distress and engage in supportive interactions can play a pivotal role in providing early emotional support, whereas unhelpful attitudes and behaviors can create barriers to help-seeking.

Theme 3: Creating a supportive environment for mental health

The need for a supportive campus environment emerged strongly across FGDs. Participants identified increasing awareness about mental health problems and help-seeking pathways as essential for promoting student mental health. They suggested strategies such as using bulletin boards to display mental health tips, organizing classroom discussions, and including mental health topics in school assemblies to improve visibility, reach a wider audience, and reduce stigma.

Participants emphasized the value of strengthening social connections to foster a culture in which students feel comfortable sharing their struggles and seeking help. Ensuring that all students feel included and valued in various campus activities was also noted as a step towards building a positive, supportive environment and reducing the sense of isolation that some students may experience. They also called for safe spaces where students can discuss mental health without fear of judgment or repercussions to normalize such discussions and facilitate access to support services.

Another subtheme was the allocation of dedicated time for mental health activities and the reduction of academic pressures. Participants suggested integrating mental health initiatives into the academic calendar to create opportunities for self-care. They also valued opportunities for personal growth and skill-building, alongside academics, as a means of fostering well-being, achievement, and purpose.

Theme 4: Student engagement and peer support

When asked about the role students could play in supporting campus mental health, participants recommended training friendly and approachable peers as mental health champions. They felt that these trained peers could raise awareness, encourage open discussion, and provide support. They emphasized that individuals selected for such roles should be naturally empathetic and respected by their peers.

A few participants proposed a peer mentoring system in which less experienced students are paired with more experienced peers. They believed that such mentoring could help mentees navigate academic and social challenges while offering mentors opportunities to develop leadership and empathy skills.

Theme 5: Access to resources for students

During the course of the group discussion, the participants were asked about the potential value of self-help materials for supporting mental health and well-being. While they generally viewed these resources as beneficial, they identified barriers to their sustained use, including difficulty maintaining motivation, fear of being judged for using such resources, and time constraints due to academic workload.

Leveraging technology for developing and disseminating self-help resources emerged as a subtheme. Participants highlighted the potential of web-based support forums and social media platforms, such as WhatsApp, to create supportive peer communities and share mental health information. They acknowledged the potential drawbacks of social media, including distraction, misinformation, and superficial engagement with content. However, they believed that creative use of digital media, such as interactive videos, images, avatars, and gamified activities, could make materials more engaging, relatable, personalized, and accessible. They felt this approach would increase student participation and interest, making students more likely to benefit from these resources.

Theme 6: Strengthening mental health services for students

Participants stressed the need for a formal, accessible counseling system to provide timely support. They recommended structured services such as regular sessions with dedicated counselors and a clear framework for accessing care.

They emphasized that confidentiality and trust are essential to encourage help-seeking. They also recommended individualized approaches to counseling to ensure that interventions meet students’ specific needs. The use of helplines was discussed, with participants noting that anonymity could encourage help-seeking. However, some expressed skepticism about their effectiveness, questioning whether helplines could provide meaningful support.

## Discussion

This study identified six key themes reflecting students’ perspectives on campus-based mental health promotion. These themes included the need for mental health initiatives tailored to students’ expressed needs, the creation of supportive environments to enhance the uptake and effectiveness of such initiatives, the role of peer support, and the importance of accessible professional services perceived as personalized and trustworthy. Self-help resources were also viewed as potentially valuable if delivered in engaging, interactive, and easily accessible formats, with technology playing an important enabling role.

The stressors students face include both personal and academic challenges, consistent with global evidence [1,2]. In the Indian context, these challenges are amplified by high family expectations for academic performance, intense competition for limited spots in prestigious courses, and uncertainty about future career paths [3,17]. This developmental phase is particularly vulnerable to mental health problems, underscoring the urgency of these concerns [4].

Participants expressed the need for responsive support systems to address stress and mental health challenges, noting that such needs are often inadequately prioritized. They emphasized the importance of orientation and sensitization programs for parents and teachers to ensure responses that are timely, empathetic, and aligned with students’ needs. These perspectives reflect, in part, generational differences that students perceived as negatively affecting their mental well-being. Parental emotional availability, or the lack thereof, has well-documented effects on youth mental health [20].

Training teachers to recognize signs of distress and provide emotional support emerged as a valued strategy. Participants stressed the need for teachers to build personal connections to perform this role effectively. Prior studies have shown that teachers are not always preferred sources of help for emotional distress, despite the substantial time students spend with them [21,22]. However, youth often value mentoring relationships that enhance motivation, provide space for open sharing, and offer emotional comfort [23].

Creating supportive campus communities in which students’ mental health needs are respected (rather than belittled or trivialized) is critically important. Such environments help students feel safe to disclose their concerns and stresses without fear of judgment, while also enabling them to receive psychological support from peers and teachers. This supportive atmosphere holds value in itself and plays an instrumental role in encouraging students to engage with available mental health services and resources. Social support is well-recognized as a protective factor for mental health and a strong facilitator of help-seeking [24-26].

Integrating mental health topics into the academic curriculum was viewed as a way to elevate mental health as a priority and embed it within the educational experience, rather than treating it as an optional add-on. This approach has been echoed in other studies [8] and may address multiple barriers to program implementation, including limited time and resources, low prioritization, poor mental health literacy, and stigma. As noted previously, mental health promotion involves shifting its position “on the scale of values held by individuals, communities, and society” [27]. Embedding such topics at all educational levels could foster more positive stakeholder attitudes toward mental health.

Participants also endorsed peer training as a strategy to foster cohesion, inclusivity, and belonging, mentor younger students, and provide first-line support for those in distress. They emphasized selecting peers who are empathetic and responsive. Research shows that peers are often the preferred source of emotional support among youth, and various studies worldwide have evaluated the role of youth engagement and peer support in mental health programs [28-31]. However, Indian literature on formal peer support systems remains limited [32,33]. The present findings suggest such initiatives may be acceptable within Indian educational contexts.

Provision of self-help resources is recognized as a helpful and scalable strategy for empowering communities to address milder problems, enhance mental health literacy, and promote appropriate help-seeking from professional resources [34]. While acknowledging these benefits for student mental health, participants in this study identified several barriers to uptake, including time constraints due to academic pressures, difficulty maintaining motivation, and stigma. They noted that uptake could be improved by fostering a supportive environment, encouraging open conversations about mental health, and prioritizing student-led awareness efforts. Participants also highlighted technology’s role in expanding access to self-help materials, particularly when resources are interactive, gamified, visually engaging, relatable, and accessible across devices. These preferences align with emerging evidence on digital mental health interventions for youth [35-39].

Social media platforms were seen as potentially useful for destigmatizing mental health and disseminating support resources, though concerns were raised about misinformation and superficial engagement. Participants advocated for a measured approach to social media use in mental health initiatives. Online supports (such as websites, chat-based platforms, and helplines) were valued for their anonymity, but opinions varied on their effectiveness. Consistent with prior studies [16,40-43], students emphasized the importance of confidentiality, minimizing the adverse social consequences of help-seeking, and ensuring access to a range of supports. They stressed the need for online and campus-based mental health services to function in a complementary fashion, while also allowing for individualized, needs-based care.

Overall, the themes converged to highlight several potentially actionable recommendations. These include: creating dedicated spaces for mental health promotion on campuses (Themes 1 and 3); conducting sensitization sessions for parents and teachers on students’ emotional needs, alongside training teachers to identify and support students in distress (Theme 2); initiating activities that enhance mental health awareness, foster open dialogue, and strengthen campus cohesion and belongingness (Theme 3); training student volunteers to promote mental health and offer peer support (Theme 4); providing access to student-friendly self-help resources and opportunities to engage with them meaningfully (Theme 5); and ensuring the availability of accessible, trustworthy, campus-based mental health services (Theme 6). Training teachers to serve as first-line support providers holds significant potential, but its feasibility, acceptability, and sustainability must be carefully examined, especially in educational settings where teachers face heavy workloads. Likewise, the shortage of qualified mental health professionals in low-resource contexts presents a major barrier to universal service access. Addressing this challenge may require a multiplicity of approaches, such as the utilization of a shared pool of service providers across campuses and the utilization of online and blended intervention formats.

Limitations

The study has certain limitations to consider when interpreting the findings. Despite efforts to recruit from diverse settings, the sample was limited to students from five urban institutions in a single district and relied on purposive sampling contingent on institutional permission. These features may limit transferability to other institutions, rural areas, other regions, or institutions with different resources and cultures. Only one FGD was conducted per campus, and groups were heterogeneous; as a result, the study was not designed or powered to compare perspectives across institution types, educational levels, or demographic subgroups, and subgroup-specific themes may have been diluted. While theme saturation did seem to emerge across the group discussions conducted, more sessions with varied groups of participants could help further confirm the same.

Methodological features of FGDs also introduce potential bias. The group format and the presence of peers may have constrained disclosure on sensitive topics, and the 45- to 60-minute session length may have limited depth. Although transcripts were translated and cross-checked, translation from Kannada to English can entail a loss of nuance. Each FGD was coded independently by a team member with peer review, and all the coders were postgraduates in psychology trained in qualitative analysis and worked under supervision. However, we did not conduct participant validation (member checking) of the final themes.

Finally, the cross-sectional design captures perspectives at a single point in time and cannot assess how recommendations translate into behavior, service uptake, or mental health outcomes. Most participating institutions lacked established counseling services, which may have shaped students’ views on the access to and nature of services; consequently, the findings primarily reflect experiences in settings with limited mental health resources.

## Conclusions

This qualitative study examined the perspectives of urban Indian secondary school and undergraduate students on campus-based mental health promotion. The findings underscore the importance of designing mental health initiatives that reflect students’ articulated priorities, such as sensitizing parents and teachers, equipping educators to recognize distress, fostering structured peer support, integrating mental health into academic calendars, and ensuring stigma-free environments with accessible, confidential services and engaging digital self-help tools. Such an alignment can enable early identification, strengthen help-seeking behaviors, and promote well-being during a formative developmental stage. An approach that actively engages and empowers key stakeholders, including teachers, students, and parents, offers significant potential in under-resourced educational and healthcare contexts. Further research in the Indian context is needed to explore differences and similarities in student perspectives across diverse gender groups and educational levels and to develop and test campus-based implementation strategies for assessing the feasibility and impact of initiatives informed by the study findings.

## Appendices

Appendix A 

**Table 4:** Focus group discussion main probes and supplementary probes Broad questions (main probes) were used first. Supplementary probes were used when the main probes did not generate sufficient discussions.

S. no.	Topic (main probes and supplementary probes)
1	What are the common challenges students face that affect their wellbeing?
	Can you please describe various kinds of difficulties or negative experiences that students commonly face, which result in stress and influence their well-being?
2	What are your views on promoting mental health and well-being in students, as is done for physical health and well-being?
	The role of physical exercises and adequate nutrition, etc., for maintaining physical health is often discussed and addressed to some extent through various activities such as sports. What about mental health and wellbeing?
	Are there some measures taken in campuses to promote mental well-being or help students deal with stress or mental health concerns?
3	Mention a few things that can be done on campuses to promote students’ mental well-being.
	What do you think are the things that educational campuses can do to support the mental well-being of students or help them cope with stress or emotional difficulties?
	What role can the parents, campus administration, and teachers play?
4	What role can students themselves play in creating an environment wherein talking about emotional difficulties is not looked down upon?
	Would it be of use to have a body of trained students who can provide a basic level of support to any student who may be undergoing significant stress or emotional difficulty?
	How could it be of help? What may be the challenges? How to overcome such challenges?
5	Would it be of any help to make information and self-help tips on various topics (e.g., nature of common mental health problems, coping with stress, when to go for professional help) available for students?
	To what extent might it be helpful?
	In what formats and how to make such resources available?
	How to ensure that such resources are actually utilized by the students?
	What about the use of informative websites and online support forums for students?
	Any other suggestions?
